# Dimensional Stability of SLA 3D-Printed Surgical Guide Resin After Steam Sterilization (121 °C and 134 °C) and Isopropyl Alcohol Disinfection: An In Vitro Study

**DOI:** 10.3390/dj14040204

**Published:** 2026-04-02

**Authors:** Ioan Sîrbu, Andreea Custura, Adelin Radu, Vlad Gabriel Vasilescu, Vladimir Nastasie, Vasile Iulian Antoniac, Marian Miculescu, Adrian Ionut Șișman, Valentin Sîrbu

**Affiliations:** 1 Department of Oral Implantology, Faculty of Dental Medicine, University of Medicine and Pharmacy “Carol Davila”, 050474 Bucharest, Romania; ioan.sirbu@umfcd.ro; 2 Department of Dental Prosthetics, Faculty of Dental Medicine, University of Medicine and Pharmacy “Carol Davila”, 050474 Bucharest, Romania; andreea-mihaela.custura@drd.umfcd.ro; 3 Faculty of Dental Medicine, University of Medicine and Pharmacy “Carol Davila”, 050474 Bucharest, Romania; vladimir.nastasie@drd.umfcd.ro; 4 Faculty Materials Science & Engineering, National University Science & Technology Politehnica Bucharest, 313 Splaiul Independentei, Dist 6, 060042 Bucharest, Romania; iulian.antoniac@upb.ro (V.I.A.); marian.miculescu@upb.ro (M.M.); 5 Faculty of Power Engineering, University Politehnica Bucharest, 060042 Bucharest, Romania; ionut_adrian.sisman@upb.ro; 6 Department of Implant-Prosthetic Therapy, Faculty of Dental Medicine, University of Medicine and Pharmacy “Carol Davila”, 050474 Bucharest, Romania; valentin.sirbu@umfcd.ro

**Keywords:** 3D printing, stereolithography, surgical guide resin, sterilization, autoclave, dimensional stability, accuracy, isopropyl alcohol

## Abstract

**Highlights:**

**What are the main findings?**

- Steam sterilization at 134 °C caused significant dimensional changes in SLA surgical guide resin.
- Sterilization at 121 °C did not significantly affect dimensional stability.
- IPA70 disinfection showed no statistically significant dimensional alterations.

**What are the implications of the main findings?**

- High-temperature sterilization may compromise accuracy of SLA-printed surgical guides.
- Lower-temperature sterilization preserves dimensional stability of Surgical Guide Resin V1.
- Sterilization protocols should be validated for guide–sleeve critical regions.

**Abstract:**

**Background**: Additively manufactured surgical guides require post-processing and subsequent decontamination prior to intraoral use. Steam sterilization and chemical disinfection protocols may influence the dimensional stability of polymer-based guide materials and potentially affect clinical fit and accuracy. **Objectives**: This in vitro study evaluated the dimensional changes of SLA 3D-printed Surgical Guide Resin V1 (Formlabs) after steam sterilization at 121 °C (AUT121) and 134 °C (AUT134) and after disinfection using 70% isopropyl alcohol (IPA70), compared with an untreated control group. **Methods**: Forty standardized specimens were fabricated using SLA technology and divided into four groups (n = 10/group): Control (CT), 121 °C steam sterilization (AUT121), 134 °C steam sterilization (AUT134), and IPA70 disinfection. Two linear measurement zones (L1 and L2) were assessed per specimen. Baseline measurements were recorded with a caliper (mm). Post-treatment measurements were obtained using microscopic evaluation, recorded in µm, and converted to mm for analysis. Dimensional change was calculated as ΔL = L_after − L_before. Within-group comparisons and between-group analyses were performed with a significance level of α = 0.05. **Results**: Steam sterilization at 134 °C (AUT 134) produced statistically significant dimensional changes in both zones (L1: *p* = 0.036; L2: *p* = 0.042). No statistically significant differences were observed in the AUT121 group (L1: *p* = 0.437; L2: *p* = 0.682) or the IPA70 group (L1: *p* = 0.164; L2: *p* = 0.086). Between-group analysis showed no significant differences for ΔL1 (*p* = 0.345), whereas ΔL2 differed significantly among groups (*p* = 0.021). **Conclusions**: Under the conditions of this study, AUT134 steam sterilization significantly affected the dimensional stability of SLA-printed Surgical Guide Resin V1 specimens. The AUT121 protocol and IPA70 disinfection did not result in statistically significant dimensional changes compared with baseline.

## 1. Introduction

Additive manufacturing has become an integral component of digital dentistry, enabling predictable fabrication of patient-specific devices such as surgical templates and guided implant components. Among the available technologies, stereolithography (SLA) provides high resolution and clinically acceptable surface quality, and it is therefore widely used for the production of surgical guides in implant dentistry.

Surgical guides are used across a broad range of clinical indications in implant dentistry, including single-tooth replacements, partially edentulous cases, and fully edentulous rehabilitations requiring multiple implant placements. Their primary function is to transfer the virtually planned implant position, angulation, and depth to the surgical site, thereby reducing intraoperative variability and improving the predictability of guided implant placement workflows. Depending on the degree of guidance provided, surgical guides may be classified as fully guided, allowing control of both drilling direction and depth, or partially guided, restricting only the drilling axis. The accuracy of a surgical guide is directly dependent on the quality of the digital planning data, the precision of fabrication, and the stability of the guide during the surgical procedure [1,2,3]. In addition to implant surgery, surgical guides and similar additively manufactured templates are employed in orthognathic surgery, oral and maxillofacial reconstruction, and orthodontic implant placement [4,5,6,7].

A variety of photopolymer resins are currently available for the fabrication of SLA-printed surgical guides, differing in their mechanical properties, biocompatibility profiles, and manufacturer-specified decontamination recommendations. Among the most widely used is Surgical Guide Resin V1 (Formlabs Inc., Somerville, MA, USA), which was selected for the present study. Other resins produced by the same manufacturer include BioMed Amber Resin (Formlabs Inc., Somerville, MA, USA), a biocompatible material intended for short-term intraoral contact and used in orofacial and dental surgical applications. Additional commercially available materials from other manufacturers offer varying degrees of translucency, rigidity, and thermal resistance, and their dimensional behavior under sterilization conditions may differ substantially from the material investigated in the present study. The selection of an appropriate resin for surgical guide fabrication should therefore consider not only its mechanical and optical properties, but also its validated response to the decontamination protocols employed in the clinical setting [8,9,10].

In clinical practice, surgical guides require post-processing steps that typically include washing, post-curing, and final decontamination. Depending on local regulations and clinical protocols, guides may undergo steam sterilization or chemical disinfection prior to intraoral use. However, thermal exposure, moisture, and chemical agents may influence polymer structure and dimensional stability, potentially affecting the accuracy and fit of the guide during guided surgical procedures [11,12,13].

In addition to accuracy requirements, infection control represents a critical aspect of the clinical workflow. Surgical guides may become contaminated during manufacturing and handling in dental laboratory environments and are subsequently used intraoperatively in contact with blood, bone, and open surgical sites. Therefore, appropriate decontamination is essential to minimize the risk of postoperative infection and to ensure patient safety. Although high-level disinfection can eliminate many microorganisms, it may not reliably inactivate bacterial spores, which supports the rationale that surgical guides should ideally be sterile during clinical use, similarly to other surgical instruments [14,15,16]. Furthermore, the classification of surgical guides as medical devices and the variability in manufacturers’ infection-control recommendations highlight the need for validated and standardized protocols that ensure both microbial safety and dimensional stability [9].

Decontamination methods differ in their bactericidal mechanisms and overall efficacy. Steam sterilization achieves microbial elimination through heat-induced protein denaturation and disruption of cellular metabolism, and is considered the most reliable method for achieving sterility, including inactivation of bacterial spores; however, thermal exposure may simultaneously compromise the mechanical and dimensional properties of sensitive polymer-based materials [17]. Chemical disinfection using alcohols such as isopropyl alcohol acts primarily through lipid membrane dissolution and protein denaturation, offering rapid broad-spectrum activity against vegetative bacteria, fungi, and enveloped viruses, although it lacks sporicidal action [18]. Regardless of the method applied, disinfection efficacy and its secondary effects on material properties should be validated individually for each material type, as surface characteristics and chemical composition may influence both microbial susceptibility and the material’s response to the decontamination agent [19]. While microbial safety remains the primary objective of decontamination protocols, the physical and dimensional consequences of these procedures on polymer-based dental materials represent a critical parallel concern, particularly for additively manufactured devices where geometric precision is clinically essential [19].

Dimensional stability is clinically relevant because even minor changes in guide geometry may translate into deviations at the implant osteotomy level, especially in fully guided workflows where tolerance levels may be limited. Surgical guides are used to transfer the virtual implant plan to the clinical site through digital planning and the integration of CBCT-based data, and their accuracy is closely linked to proper fit, stability, and rigidity during the surgical procedure [1,2,3,20,21]. Several studies have investigated the influence of steam sterilization on the accuracy and fit of 3D-printed surgical guides and reported that dimensional changes may occur depending on material properties and sterilization parameters; however, clinically acceptable outcomes may still be achieved under specific conditions. Consequently, identifying the impact of commonly used sterilization and disinfection protocols on 3D-printed surgical guide materials remains essential for safe and predictable clinical implementation. This is particularly relevant in guided workflows, where the guide–sleeve region plays a critical role in transferring drilling position and angulation [9,21,22,23,24,25,26,27].

The aim of this in vitro study was to evaluate the dimensional changes of SLA 3D-printed Surgical Guide Resin V1 (Formlabs) after steam sterilization at 121 °C (AUT121) and 134 °C (AUT134), and after disinfection using 70% isopropyl alcohol (IPA70), compared to an untreated control group. The null hypothesis was that no significant dimensional differences would be observed between baseline and post-treatment measurements across the tested protocols.

## 2. Materials and Methods

### 2.1. Specimen Design

**Figure 1:**
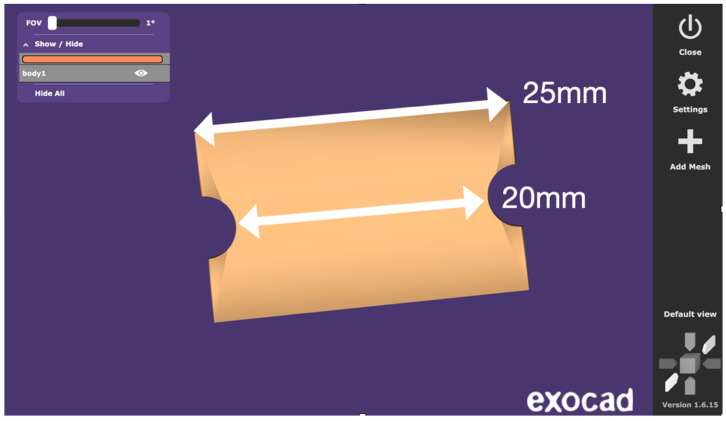
Digital design of the specimen with annotated linear dimensions (length of the entire piece—L1, and length between the two semi-circular concavities simulating the sleeve geometry—L2).

**Figure 2:**
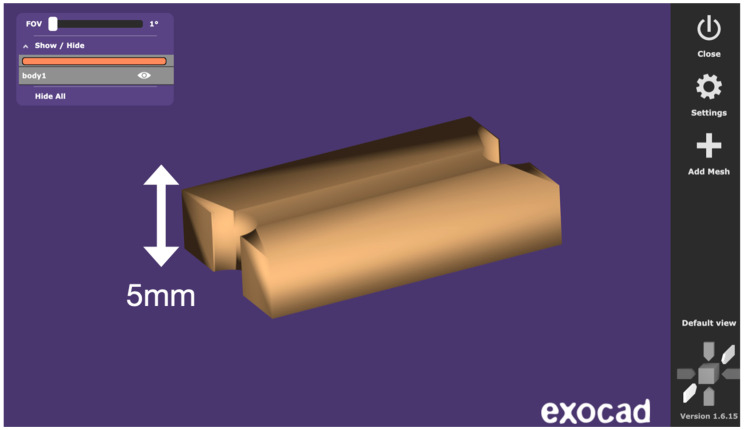
Digital design of the specimen with annotated linear dimensions (height).

**Figure 3:**
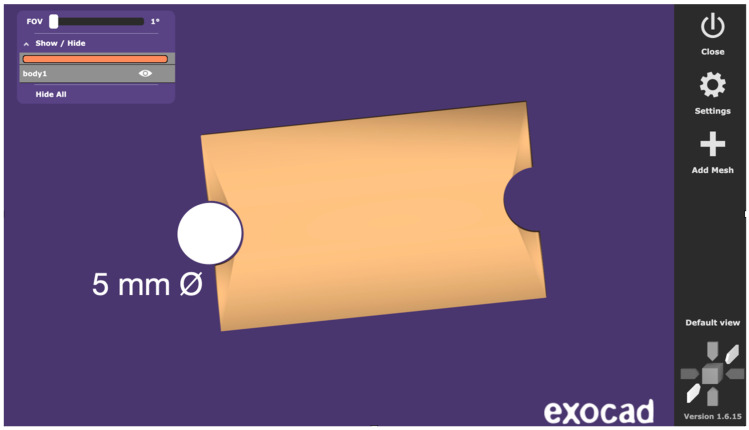
Digital design of the specimen showing the circular feature simulating the sleeve geometry.

Standardized test specimens were designed as rectangular blocks measuring 25 mm in length, 20 mm in length between the two semi-circular concavities simulating the sleeve geometry, 15 mm in width, and 5 mm in height. Each specimen incorporated a semi-circular concavity positioned at the midpoint of both lateral faces. Together, these paired concavities reproduced the geometry of a cylindrical sleeve housing, mimicking the recess typically present in stackable surgical guides for implant placement. This feature provided a clinically relevant region for evaluating dimensional stability following sterilization. The central axis of the block and the sleeve recess served as fixed reference landmarks during all dimensional assessments (Figure 1, Figure 2 and Figure 3).

The specimen geometry was purposefully designed for dimensional stability assessment rather than mechanical testing, and therefore does not follow a standardized ISO mechanical testing norm. The overall length of 25 mm was selected to provide a measurement span sufficient to detect dimensional changes, as linear measurements exceeding 10 mm allow for more reliable detection of small deformations compared to shorter or cubic geometries used in some prior studies. While simpler shapes such as cubes or rectangular bars have been employed in previous investigations of sterilization-related dimensional changes, these designs offer limited geometric complexity and do not reproduce clinically relevant features of surgical guide components. The specimen design used in the present study was informed by a review of existing methodologies and was intentionally developed to incorporate two distinct measurement zones with differing geometric contexts. The overall length (L1) represents a simple linear reference across the full specimen, while the distance between the paired semi-circular concavities (L2) represents the sleeve-related region, where the two opposed semicircles together form a full circle simulating the cylindrical recess of an implant sleeve housing. This dual-zone approach allows simultaneous assessment of global and locally constrained dimensional behavior, offering greater clinical relevance than single-zone rectangular or cubic specimens.

The sample size of 10 specimens per group (n = 10) was determined based on precedent from comparable in vitro studies evaluating dimensional changes of 3D-printed dental materials following sterilization and disinfection protocols, in which group sizes ranging from 5 to 10 specimens have been reported. A formal a priori power calculation was not performed; however, a minimum of 5 specimens per group is generally considered acceptable for this type of dimensional stability assessment, and the selected sample size of n = 10 was chosen to increase statistical power, reduce the influence of individual specimen variability, and improve the reliability of both within-group and between-group comparisons. This sample size is consistent with those reported in similar in vitro investigations in the field [8,9,10,15,28].

### 2.2. 3D Printing Protocol

**Figure 4:**
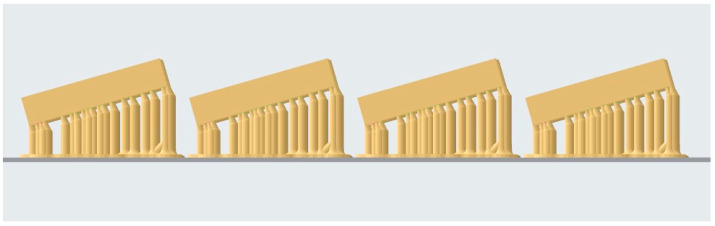
Digital design of the specimen showing the 16° orientation angle.

All specimens were fabricated using a Form 3B+ stereolithographic (SLA) 3D printer (Formlabs Inc., Somerville, MA, USA) with Surgical Guide Resin V1. The Form 3B+ employs Low Force Stereolithography (LFS) technology, utilizing a single Light Processing Unit (LPU) with a 405 nm wavelength laser, a power output of 250 mW, and an 85 μm laser spot size, certified as a Class 1 laser product (EN 60825-1). The 405 nm violet laser selectively polymerizes the photosensitive resin layer by layer through a flexible resin tank, which reduces peel forces during printing and contributes to improved dimensional accuracy. During printing, each specimen was oriented at a 16° angle with a layer thickness of 0.1 mm. A total of 40 specimens were produced following identical fabrication parameters (Figure 4).

**Figure 5:**
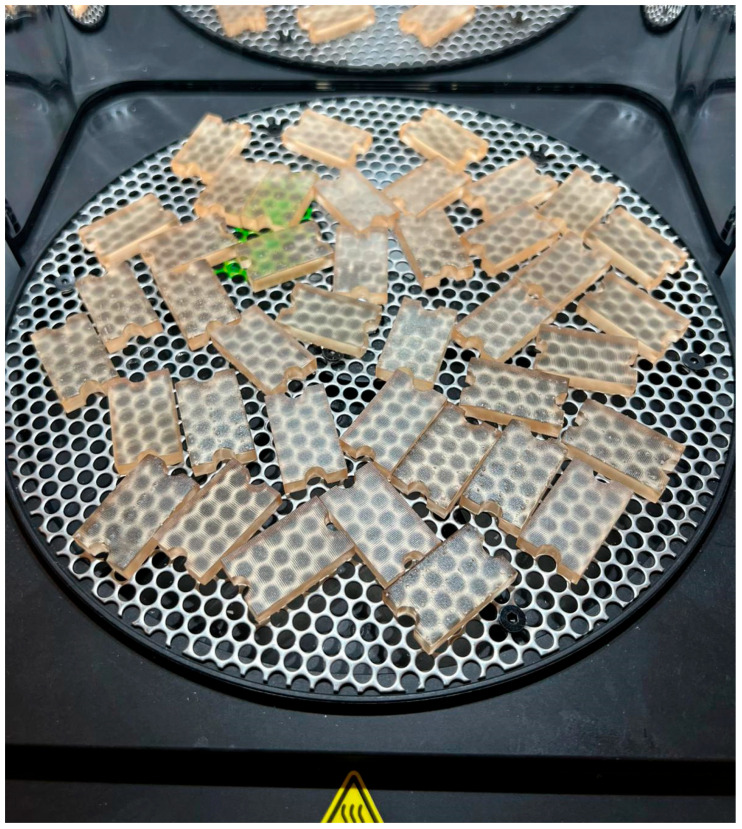
Post-curing of the printed specimens in the Form Cure unit using the manufacturer’s dedicated program for Surgical Guide Resin V1.

Immediately after printing, all specimens were washed in isopropyl alcohol (IPA) to remove residual uncured resin. Post-curing was performed in the Form Cure unit using the manufacturer’s dedicated program for Surgical Guide Resin V1, ensuring complete polymerization under standardized temperature and light conditions at 405 nm (Figure 5).

### 2.3. Experimental Groups and Sterilization Protocols

The 40 specimens were randomly allocated into four groups (n = 10/group):

- Group 1 (n = 10): CT;
- Group 2 (n = 10): AUT121;
- Group 3 (n = 10): AUT134;
- Group 4 (n = 10): IPA70.

Autoclave sterilization was performed using the Yeson Daslav Touch autoclave ((Ningbo Haishu Yeson Medical Device Co., Ltd., Ningbo, China), employing the universal sterilization cycle for both the 121 °C and 134 °C groups.

### 2.4. Dimensional Measurements

**Figure 6:**
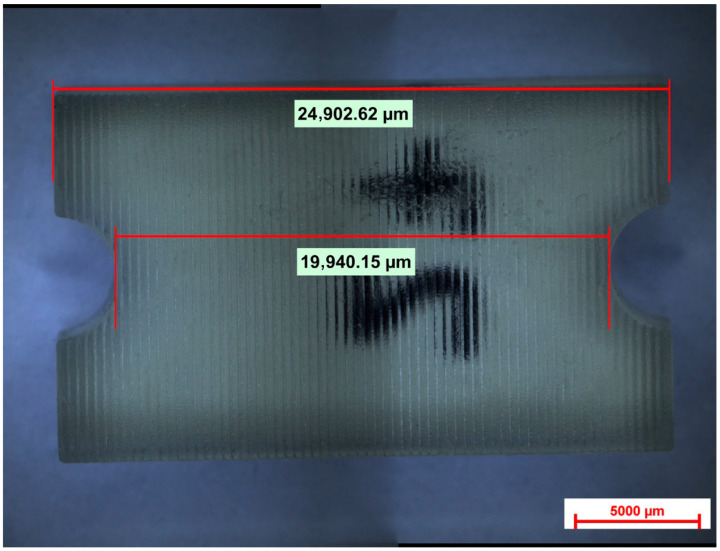
Printed specimen showing the two predefined measurement zones (L1 and L2) corresponding to the overall length of the piece and the margins of the sleeve-like recess.

Two clinically relevant measurement zones were defined on each specimen (L1 and L2) and were located on opposite lateral surfaces, corresponding to the overall length of the piece and to the margins of the sleeve-like recess created by the paired semi-circular concavities. These zones were selected to represent the region most sensitive to dimensional alterations in stackable surgical guide components (Figure 6).

Baseline measurements (Before) were recorded for all specimens using a caliper and expressed in millimeters (mm). After completion of the assigned sterilization/disinfection protocol, all specimens were re-measured using a digital caliper (precision 0.01 mm) as an additional control measurement. Subsequently, post-treatment measurements (After) were obtained using microscopic evaluation (Nikon Eclipse MA100N, 0.63×; (Nikon Corporation, Tokyo, Japan)), and dimensional values were recorded in micrometers (µm) and converted to millimeters (mm) for statistical analysis. For each specimen and measurement zone, dimensional change was calculated using the following equation: ΔL = L_after − L_before, where L_after corresponds to the microscope-based post-treatment value converted to mm and L_before corresponds to the baseline caliper measurement.

### 2.5. Statistical Analysis

For inferential statistics and *p*-value calculation, dimensional change was computed using the microscope-based post-treatment values converted to mm (ΔL = L_after (microscope) − L_before (caliper)). Caliper-based post-treatment measurements were not included in the primary statistical analysis.

All measurements were recorded and processed in Microsoft Excel (Microsoft Corp., Redmond, WA, USA). Microscope measurements originally obtained in micrometers (µm) were converted to millimeters (mm) by dividing by 1000. Dimensional changes were calculated for each specimen and measurement zone using the formula: ΔL = L_after − L_before.

Normality was assessed prior to inferential testing. Paired comparisons between baseline and post-treatment measurements within each experimental group were performed using the paired Student’s *t*-test. Non-parametric confirmation was performed using the Wilcoxon signed-rank test. Between-group comparisons of dimensional change (ΔL) were assessed using one-way analysis of variance (ANOVA). Post hoc multiple comparisons were performed using Tukey’s test. The significance level was set at α = 0.05.

## 3. Results

Paired comparisons between baseline (caliper) and post-treatment (microscope) measurements revealed protocol-dependent dimensional changes. In the 121 °C group, no statistically significant differences were identified for either measurement zone (L1: *p* = 0.437; L2: *p* = 0.682). Similarly, the IPA70 group showed no significant changes (L1: *p* = 0.164; L2: *p* = 0.086). In contrast, the 134 °C sterilization group exhibited statistically significant dimensional reductions in both zones (L1: *p* = 0.036; L2: *p* = 0.042). The control group demonstrated no significant change in L1 (*p* = 0.384), while a statistically significant increase was observed for L2 (*p* = 0.030).

Between-group analysis of dimensional change (ΔL) demonstrated no significant differences among groups for ΔL1 (one-way ANOVA, *p* = 0.345). However, ΔL2 differed significantly among groups (one-way ANOVA, *p* = 0.021). Tukey post hoc analysis for ΔL2 identified a statistically significant difference between control and IPA70 (*p* = 0.020), while other pairwise comparisons were not statistically significant (Table 1 and Table 2).

## 4. Discussion

The null hypothesis of this study stated that no significant dimensional differences would be observed between baseline and post-treatment measurements across the tested decontamination protocols. Based on the results obtained, the null hypothesis was partially rejected. Statistically significant dimensional changes were identified in the Aut134 group for both measurement zones (ΔL1: *p* = 0.036; ΔL2: *p* = 0.042), indicating that steam sterilization at 134 °C induced measurable dimensional alterations in SLA-printed Surgical Guide Resin V1 specimens. In contrast, the null hypothesis was not rejected for the Aut121 group (ΔL1: *p* = 0.437; ΔL2: *p* = 0.682) and the IPA70 group (ΔL1: *p* = 0.164; ΔL2: *p* = 0.086), as no statistically significant dimensional changes were detected. A statistically significant change was also observed for ΔL2 in the CT group (*p* = 0.030), suggesting that time-dependent dimensional variability may occur independently of any decontamination procedure, thereby contributing to the partial rejection of the null hypothesis and highlighting the relevance of the untreated control condition.

The present in vitro study evaluated the dimensional stability of SLA 3D-printed Surgical Guide Resin V1 (Formlabs) following two steam sterilization protocols (121 °C and 134 °C) and a chemical disinfection protocol (IPA70), compared to a control group. The primary finding was that steam sterilization at 134 °C resulted in statistically significant dimensional changes in both measurement zones (L1 and L2), whereas the 121 °C cycle and IPA70 disinfection did not produce significant deviations. These findings indicate that higher-temperature sterilization exerts a measurable effect on polymer-based guide materials, which is particularly relevant in guided surgery where small geometric deviations may compromise fit and drilling accuracy [2,8,14,15,28,29,30,31,32].

The statistically significant reduction in both L1 and L2 after 134 °C sterilization suggests that this protocol induces dimensional instability, likely through thermally driven mechanisms such as post-curing, stress relaxation, or polymer deformation. This interpretation is consistent with previous studies reporting that sterilization parameters influence the accuracy of printed surgical guides depending on material composition and processing conditions. Clinically, alterations at sleeve-related landmarks are critical, as the guide–sleeve–drill relationship represents a key determinant of positional accuracy in fully guided implant placement.

In contrast, the 121 °C sterilization cycle did not produce statistically significant differences compared with baseline, indicating a more dimensionally stable behavior under lower thermal load. This observation aligns with prior reports showing that lower-temperature sterilization protocols may maintain dimensional integrity within clinically acceptable limits, whereas higher temperatures increase the risk of material alteration depending on geometry and resin characteristics. The absence of significant changes at 121 °C is therefore likely related to reduced thermal stress and lower susceptibility to polymer distortion.

The IPA70 disinfection protocol did not result in statistically significant dimensional changes, although a trend toward reduction was observed. Chemical exposure may induce transient swelling or minor surface modifications depending on exposure time and resin composition, without necessarily producing detectable linear deviations. It should be emphasized that disinfection does not replace sterilization, and clinical decisions must consider infection-control requirements. Nevertheless, IPA-based protocols remain widely used due to their practicality, and their dimensional impact remains relevant when thermal sterilization is avoided [9,10,15,20,28,30,31,32,33,34].

Beyond dimensional changes, both chemical and thermal decontamination protocols may affect the physical properties of polymer-based materials. Exposure to alcohols and other disinfectants has been associated with changes in surface roughness, wettability, and microhardness, as well as polymer matrix degradation through solvent absorption and plasticization [19]. In SLA-printed resins, these effects may be amplified by residual monomer content, ongoing post-curing reactions, and environmental aging [28]. Consequently, mechanical behavior may be altered even in the absence of measurable dimensional changes. The statistically significant ΔL2 variation observed in the CT group may reflect such intrinsic time-dependent material dynamics. Therefore, dimensional assessment alone may underestimate the overall impact of decontamination protocols, and future studies should incorporate mechanical and surface characterization alongside geometric evaluation [9,10].

Between-group analysis demonstrated a statistically significant difference for ΔL2 but not for ΔL1, indicating a region-dependent dimensional response. This finding is consistent with the heterogeneous geometry of surgical guides, where variations in thickness, curvature, and residual stress distribution may influence local deformation under thermal or chemical exposure [8,9,14,15,35]. Additionally, sleeve-related concavities may introduce localized constraints that promote non-uniform dimensional changes. Accordingly, multi-zone evaluation may provide a more clinically meaningful assessment than single linear measurements [10,20,21,29,33,36].

The susceptibility of dental polymers to aging-related changes is well documented. Polyamide 12 (PA12) has been shown to exhibit reduced modulus of elasticity after thermal and mechanical aging, as well as increased surface roughness and discoloration following chemical exposure [37]. These findings demonstrate that material behavior is influenced not only by composition but also by manufacturing technique. By analogy, SLA-printed photopolymer resins may undergo changes in mechanical and surface properties beyond linear dimensional deviation, reinforcing the need for material-specific validation of decontamination protocols.

This study introduces several relevant contributions. It focuses on a clinically used material (Surgical Guide Resin V1), evaluates multiple decontamination protocols within a standardized framework, and incorporates a specimen design that includes a sleeve-simulating region of functional relevance. Additionally, the use of two measurement zones enabled detection of region-dependent behavior, while the identification of dimensional changes in the untreated control group highlights the role of intrinsic material variability over time [8,9,10,14,15,28].

A limitation of this study is the use of different measurement modalities for baseline and post-treatment assessment (caliper vs. microscope), which may introduce systematic bias despite increased sensitivity of microscopic evaluation. Future studies should standardize measurement techniques and incorporate full-surface deviation analyses, such as 3D scanning and superimposition, to improve accuracy and reproducibility [1,6,9,34].

Overall, the findings demonstrate that sterilization parameters, particularly high-temperature steam sterilization at 134 °C, can influence the dimensional stability of Surgical Guide Resin V1. Given the direct relationship between guide accuracy and clinical outcomes in guided implant surgery, careful selection and validation of sterilization protocols are essential [32,33].

This study has several limitations. The use of simplified specimen geometry may limit extrapolation to full clinical guides. Differences in measurement methodology may introduce variability. The analysis was restricted to linear measurements in two zones and did not include full 3D surface assessment. Additionally, the sample size (n = 10 per group) may limit statistical power and does not account for variability related to manufacturing or long-term aging.

## 5. Conclusions

Within the limitations of this in vitro study, the following conclusions can be drawn:

1. “Steam sterilization at 134 °C (Aut134) resulted in statistically significant dimensional changes in SLA 3D-printed Surgical Guide Resin V1 (Formlabs) specimens in both evaluated zones (ΔL1 and ΔL2), indicating that this protocol may compromise the dimensional integrity of this specific resin material and should be used with caution in clinical workflows requiring high geometric accuracy.”

2. “Steam sterilization at 121 °C (Aut121) did not produce statistically significant dimensional changes compared with baseline, suggesting that lower-temperature autoclave cycles may represent a dimensionally safer decontamination option for this material when steam sterilization is clinically indicated.”

3. “IPA70 disinfection did not result in statistically significant dimensional changes, although a tendency toward dimensional reduction was observed. Chemical disinfection with IPA70 may therefore be considered a viable alternative from a dimensional stability standpoint, while acknowledging that disinfection does not achieve the same level of microbial safety as sterilization.”

4. “Between-group comparisons indicated that dimensional change was zone-dependent, with significant differences detected for ΔL2 but not for ΔL1, highlighting that different regions of a surgical guide component may respond differently to the same decontamination protocol. This finding underscores the importance of evaluating multiple clinically relevant measurement zones rather than relying on a single linear assessment.

From a clinical perspective, these results suggest that the choice of decontamination protocol for SLA-printed surgical guides should be material-specific and validated prior to routine clinical application, particularly in fully guided implant workflows where dimensional accuracy directly influences implant positioning. Clinicians and dental laboratories are encouraged to consider lower-temperature sterilization or validated chemical disinfection protocols when working with Surgical Guide Resin V1, and to avoid routine use of 134 °C steam sterilization for this material without prior dimensional verification

Future research should investigate the cumulative effect of repeated sterilization cycles on dimensional stability, incorporate full-surface 3D deviation analysis using digital scanning and best-fit superimposition, and extend evaluations to other commercially available surgical guide resins and alternative decontamination methods. Long-term studies assessing the combined influence of sterilization, mechanical loading, and aging on the clinical performance of additively manufactured surgical guides would further strengthen the evidence base for validated infection-control protocols in guided implant surgery.

## Figures and Tables

**Table 1:** Dimensional changes (Δ, mm) at measurement zone L1 (Δ L1) following steam sterilization at 121 °C and 134 °C, chemical disinfection (IPA70), and control conditions. Data are presented as mean ± standard deviation.

Group	n	Mean ΔL1 (mm)	SD	*p*-Value (vs. Baseline)	Significance
CT	10	+0.036	0.124	0.384	ns
Aut121	10	−0.057	0.222	0.437	ns
Aut134	10	−0.072	0.092	0.036	*
IPA70	10	−0.039	0.082	0.164	ns

Between-group comparison (one-way ANOVA): *p* = 0.345 (ns). ns = not significant (*p* > 0.05); * = significant (*p* ≤ 0.05).

**Table 2:** Dimensional changes (Δ, mm) at measurement zone L2 (Δ L2) following steam sterilization at 121 °C and 134 °C, chemical disinfection (IPA70), and control conditions. Data are presented as mean ± standard deviation.

Group	n	Mean ΔL2 (mm)	SD	*p*-Value (vs. Baseline)	Significance
CT	10	+0.145	0.178	0.030	*
Aut121	10	+0.031	0.232	0.682	ns
Aut134	10	−0.041	0.055	0.042	*
IPA70	10	−0.079	0.129	0.086	ns

Between-group comparison (one-way ANOVA): *p* = 0.021 (significant) Tukey post hoc: CT vs. IPA70, *p* = 0.020 (*). ns = not significant (*p* > 0.05); * = significant (*p* ≤ 0.05).

## Data Availability

The original contributions presented in this study are included in the article. Further inquiries can be directed to the corresponding authors.
